# The Cholesterol-Dependent Cytolysin Pneumolysin from *Streptococcus pneumoniae* Binds to Lipid Raft Microdomains in Human Corneal Epithelial Cells

**DOI:** 10.1371/journal.pone.0061300

**Published:** 2013-04-05

**Authors:** Sidney D. Taylor, Melissa E. Sanders, Nathan A. Tullos, Stephen J. Stray, Erin W. Norcross, Larry S. McDaniel, Mary E. Marquart

**Affiliations:** Department of Microbiology, University of Mississippi Medical Center, Jackson, Mississippi, United States of America; Instituto Butantan, Brazil

## Abstract

*Streptococcus pneumoniae* (pneumococcus) is an opportunistic bacterial pathogen responsible for causing several human diseases including pneumonia, meningitis, and otitis media. Pneumococcus is also a major cause of human ocular infections and is commonly isolated in cases of bacterial keratitis, an infection of the cornea. The ocular pathology that occurs during pneumococcal keratitis is partly due to the actions of pneumolysin (Ply), a cholesterol-dependent cytolysin produced by pneumococcus. The lytic mechanism of Ply is a three step process beginning with surface binding to cholesterol. Multiple Ply monomers then oligomerize to form a prepore. The prepore then undergoes a conformational change that creates a large pore in the host cell membrane, resulting in cell lysis. We engineered a collection of single amino acid substitution mutants at residues (A370, A406, W433, and L460) that are crucial to the progression of the lytic mechanism and determined the effects that these mutations had on lytic function. Both Ply^WT^ and the mutant Ply molecules (Ply^A370G^, Ply^A370E^, Ply^A406G^, Ply^A406E^, Ply^W433G^, Ply^W433E^, Ply^W433F^, Ply^L460G^, and Ply^L460E^) were able to bind to the surface of human corneal epithelial cells (HCECs) with similar efficiency. Additionally, Ply^WT^ localized to cholesterol-rich microdomains on the HCEC surface, however, only one mutant (Ply^A370G^) was able to duplicate this behavior. Four of the 9 mutant Ply molecules (Ply^A370E^, Ply^W433G^, Ply^W433E^, and Ply^L460E^) were deficient in oligomer formation. Lastly, all of the mutant Ply molecules, except Ply^A370G^, exhibited significantly impaired lytic activity on HCECs. The other 8 mutants all experienced a reduction in lytic activity, but 4 of the 8 retained the ability to oligomerize. A thorough understanding of the molecular interactions that occur between Ply and the target cell, could lead to targeted treatments aimed to reduce the pathology observed during pneumococcal keratitis.

## Introduction

*Streptococcus pneumoniae* (pneumococcus) is a worldwide pathogen responsible for both invasive and noninvasive infections, including pneumonia, meningitis, bacteremia, and otitis media [1]–[5]. Additionally, pneumococcus is known to be the etiologic agent of several ocular infections including conjunctivitis, endophthalmitis, and keratitis [6]–[14]. Pneumococcus is typically among the top three most commonly isolated species from cases of bacterial keratitis, an infection of the cornea of the eye [13], [15], [16]. Pneumococcal keratitis can be a sight-threatening infection if left untreated or if treatment is delayed. Corneal ulceration occurs during the course of the infection and often results in an opaque scarification of the corneal surface after the infection is cleared. The lytic action of pneumolysin (Ply), a 53 kilodalton (kDa) virulence protein produced by *S. pneumoniae*, is responsible for the formation of corneal ulcers and is a major contributor to pneumococcal virulence as a whole [17]–[23]. Ply is a member of the cholesterol-dependent cytolysin (CDC) family of proteins, a group of pore-forming proteins from several gram positive bacterial genera including *Streptococcus, Listeria, Bacillus,* and *Clostridium* [24]. All CDCs are thought to share a common lytic mechanism which is 100% dependent on the presence of cholesterol in the target cell membrane [24]. In the case of Ply, cholesterol serves as the initial binding ligand which anchors Ply to the host cell surface [25]. After binding to cholesterol, surface-bound Ply monomers are oriented perpendicular to the cell surface and begin to diffuse laterally across the host membrane [26]. Eventually Ply monomers will interact with one another and oligomerize to form a large multimeric prepore structure consisting of 34–50 monomers [24], [27]. The prepore structure then undergoes a synchronized conformational change that causes a vertical collapse of the entire complex and the insertion of two β-hairpin structures from each monomer into the host membrane [28]. The β-hairpins collectively form a large β-barrel pore approximately 25 nm in diameter that traverses the cell membrane resulting in osmotic disregulation and cell death [28], [29].

Much of what is known about Ply has been extrapolated from previous findings focusing on the lytic mechanism of perfringolysin (Pfo), the CDC produced by *Clostridium perfringens.* The tertiary structure of domain 4 of Pfo contains 4 peptide loops that were found to directly enter the lipid environment upon interaction with cholesterol containing membranes [26]. One of these loops is commonly referred to as the undecapeptide sequence and was originally hypothesized to interact directly with cholesterol and facilitate membrane anchoring since the sequence is highly conserved in most CDCs [30], [31]. However, intermedilysin (Ily), of *Streptococcus intermedius*, has been found to have an altered undecapeptide sequence which directly targets human CD59 (protectin) as the initial binding target [32]. Despite the modified undecapeptide, Ily is similar to Pfo in that it still contains the other 3 hydrophobic loops, commonly referred to as L1–L3, and these L1–L3 loops enter the lipid environment of cholesterol-containing membranes in the same manner as seen in Pfo. The behavior of the L1–L3 loops indicates that one or more of these loops likely interact with cholesterol in the host membrane [33]. Recently, two residues found within the L1 loop (T490 and L491 in Pfo) have been proposed to be the cholesterol recognition motif for all CDCs, as the two residues are 100% conserved across the CDC family and mutagenesis of these two residues results in drastic reductions in cholesterol binding [34].

Within the host cell membrane, cholesterol is found at a higher concentration within self-segregating regions known as lipid raft microdomains [35], [36]. When compared to the surrounding phospholipid membrane, also known as the liquid disordered phase (L_d_), lipid rafts typically contain a higher concentration of sphingolipids intercalated with cholesterol [35], [36]. Numerous studies suggest that the segregation of lipid raft microdomains from the surrounding bilayer functions to compartmentalize or concentrate specific proteins in order to perform cellular functions, including endocytosis and intracellular signaling by second messenger pathways [37]–[40]. Since cholesterol serves as the initial binding target for Ply, it is hypothesized that Ply will localize and interact with lipid raft microdomains on the host cell surface. Furthermore, lipid rafts may function to congregate Ply monomers and increase the probability of oligomerization.

Traditionally, the Ply mechanism of action has been studied using either human red blood cells or synthetic membranes, but Ply has never been examined using cells of the human corneal epithelium, which are an example of a cell that is directly and measurably affected by Ply during the course of an infection. Investigating the lytic mechanism of Ply in the context of ocular pathogenesis could lead to the development of targeted treatments that aim to prevent the pathology caused by Ply during pneumococcal keratitis.

## Materials and Methods

### Structural Diagrams

Ply structural cartoons were generated using MacPyMOL version 1.3 based on the primary sequence of Ply from the *S. pneumoniae* D39 genome (GenBank Accession Number CP000410.1).

### Plasmids, Bacterial Strains, and Cell Growth Conditions

The wild-type *ply* gene (ply^WT^) was amplified from the chromosomal DNA of *S. pneumoniae* D39, and cloned into the pET101D expression vector (Invitrogen, Carlsbad, CA) as previously described and was subsequently named pET101D-*ply* ^WT^ [41]. All recombinant Ply expression vectors were transformed in Escherichia coli BL21, and grown at 37°C with aeration at 200 rpm in Luria-Bertani medium (LB) containing 50 μg/ml carbenicillin.

Human corneal epithelial cells (HCECs) were a kind gift from Dr. Haydee Bazan (Louisiana State University Eye Center, New Orleans, LA) and were originally established by Dr. Roger Beuerman (Singapore Eye Research Institute). The first use of the HCEC line was described by Sharma et al. in 2003 [42]. HCECs were cultivated in serum-free Clonetics keratinocyte media (KGM; Clonetics BioWhittaker Europe, Verviers, Belgium) supplemented with growth factors and antibiotics as previously described [42], [43].

### Site-Directed Mutagenesis

Single amino acid substitutions were introduced into the pET101D-*ply* ^WT^ vector, specifically A370G, A370E, A406G, A406E, W433G, W433E, L460G, and L460E. The pET101D-*ply* ^W433F^ vector was previously engineered by Thornton and McDaniel using the Genetailor site-directed mutagenesis kit (Invitrogen, Carlsbad, CA), and the same methodology was used to engineer the other mutants with the exception of pET101D-*ply* ^A406G^ and pET101D-*ply* ^A406E^ [41]. Both A406 mutants were engineered using PCR QuikChange site-directed mutagenesis (Stratagene, La Jolla, CA). Custom primers were designed for each mutagenesis reaction (Table 1).

**Table 1:** Oligonucleotide Primers for Mutagenesis.

	Primer	Sequence (5′–3′)	Study
1	Ply^A370G^ For	ctgctggatcatagtggtggctatgttgcccaa	This Study
2	Ply^A370G^ Rev	accactatgatccagcagtaaatctccgtttct	This Study
3	Ply^A370E^ For	ctgctggatcatagtggtgaatatgttgcccaa	This Study
4	Ply^A370E^ Rev	accactatgatccagcagtaaatctccgtttct	This Study
5	Ply^A406G^ For	ggcaggatttgacgggtcactttaccactag	This Study
6	Ply^A406G^ Rev	ctagtggtaaagtgacccgtcaaatcctgcc	This Study
7	Ply^A406E^ For	ggcaggatttgacggagcactttaccactag	This Study
8	Ply^A406E^ Rev	ctagtggtaaagtgctccgtcaaatcctgcc	This Study
9	Ply^W433G^ For	gagtgtaccgggcttgccggggaatggtggcgt	This Study
10	Ply^W433G^ Rev	ggcaagcccggtacactctctaattttgac	This Study
11	Ply^W433E^ For	gagtgtaccgggcttgccgaggaatggtggcgt	This Study
12	Ply^W433E^ Rev	ggcaagcccggtacactctctaattttgac	This Study
13	Ply^W433F^ For	gagtgtaccgggcttgccttcgaatggtggcgt	Thornton and McDaniel, 2005 [41]
14	Ply^W433F^ Rev	ggcaagcccggtacactctctaattttgac	Thornton and McDaniel, 2005 [41]
15	Ply^L460G^ For	tctatttggggaacaactggctatcctcaggta	This Study
16	Ply^L460G^ Rev	agttgttccccaaatagaaatcgtccgctt	This Study
17	Ply^L460E^ For	tctatttggggaacaactgaatatcctcaggta	This Study
18	Ply^L460E^ Rev	agttgttccccaaatagaaatcgtccgctt	This Study

Custom overlapping primers were designed to introduce site-directed mutations into the coding sequence of Ply.

### Recombinant Pneumolysin Purification

*E. coli* BL21 was grown in LB plus 50 µg/ml carbenicillin to an OD_600_ of 0.5, which corresponds to 1x10^9^ cfu/ml. Isopropyl-β-D-thiogalactopyranoside (IPTG) was added to the culture to a final concentration of 1 mM to induce Ply expression. The culture was grown for an additional 4–5 h. Bacterial cells were collected by centrifugation for 10 min at 8,000 x g and 4°C. The remainder of the purification was performed on ice. The bacterial pellet was suspended in 40 ml of extraction/wash buffer (50 mM sodium phosphate, 300 mM sodium chloride) and lysed by sonication (5 intervals; 30 sec per interval, with alternating 30 sec rest periods). The cellular debris was removed by centrifugation for 20 min at 20,000 x g and 4°C. Ply was purified from the soluble intracellular contents by Talon metal affinity resin chromatography (BD Biosciences, Franklin Lakes, NJ), which selectively binds to the 6X histidine tag added by the pET101D expression vector. After elution from the resin, Ply was extensively dialyzed in 4 L of phosphate-buffered saline (PBS; 150 mM sodium chloride, 2.3 mM monobasic sodium phosphate, 7.7 mM dibasic sodium phosphate, pH 7.2). Purified Ply was confirmed via SDS-PAGE as a single 53 kDa band (data not shown), and was quantified by detecting the absorbance at 280 nm with an extinction coefficient of 72,000 cm^−1^M^−1^.

### Cytotoxicity Assay

Ply cytotoxicity on HCECs was measured using the Live/Dead cytotoxicity kit (Invitrogen, Carlsbad, CA). HCECs were grown to confluency in 96-well tissue culture plates. Each monolayer was washed twice with PBS and labeled with various concentrations of either Ply^WT^ or mutant Ply for 3 h at 37°C. The cells were washed with PBS twice and labeled with a final concentration of 2 µM calcein AM, a selective intracellular label for healthy cells. The cells were placed in the dark for 30 min at 37°C. The cells were then washed with PBS, and fluorescence was detected by fluorescence spectrometry at 530 nm. Triton X-100 (TX100) treated HCECs served as the positive control representing 100% lysis, and PBS treated cells served as the negative control. Each experiment was performed in triplicate, and relative percent survival was calculated as (A_sample_−A_TX100_)/(A_PBS_−A_TX100_)×100. Data was presented as relative percent survival.

### Hemolysis Assay

Various concentrations (480–0.2 nM) of Ply^WT^ or mutant Ply were incubated with 2% rabbit red blood cells (RBCs) in a round bottom 96-well plate for 30 min at 37°C. The plate was centrifuged for 5 min at 700 x g to collect any intact RBCs. The supernatant from each well was transferred to a flat-bottom 96-well plate and RBC lysis was quantified based on hemoglobin concentration in the supernatant. Hemoglobin was quantified by measuring absorbance at 450 nm by spectrophotometry. Each assay was performed in triplicate. RBCs and 1% TX100 served as the positive control and represents 100% lysis. All results are presented as percent hemolysis relative to the positive control. Relative percent hemolysis was calculated as (A_sample_−A_PBS_)/(A_TX100_−A_PBS_)×100. Rabbit RBCs were collected from New Zealand white rabbits (Charles River, Wilmington, MA). Our protocol was approved by and the rabbits were maintained according to the guidelines of the University of Mississippi Medical Center Institutional Animal Care and Use Committee (IACUC) and the tenets of the Association for Research in Vision and Ophthalmology (ARVO) Resolution on the Use of Animals in Ophthalmic and Vision Research. Rabbits were anesthetized by intramuscular injection of 100 mg of ketamine prior to drawing blood via ear vein. The rabbits were provided enrichment items for entertainment during housing. The animals were not euthanized at the end of the study.

### Western Blot (Oligomerization Assay)

The oligomerization assay protocol was modified from a previously published protocol performed by Soltani *et al.* [33]. Five hundred ng of Ply^WT^ or mutant Ply was incubated either in the presence or absence of 10^6^ HCECs for 30 min at 37°C in PBS to a total volume of 20 μl. Each cell suspension was mixed 1∶1 with 2X SDS Loading Buffer (100 mM Tris-HCl, 200 mM dithiothreitol, 4% w/v SDS, 0.2% bromophenol blue, 20% v/v glycerol) and was subjected to 6% SDS-PAGE. The gel was then electroblotted to a PVDF membrane, and blocked in 5% skim milk in NP-40 buffer (50 mM Tris-HCl, 150 mM NaCl, 5 mM EDTA, 0.05% v/v Nonidet P-40) for 1 h at 25°C. The membrane was rinsed with NP-40 buffer and then labeled with rabbit polyclonal anti-Ply serum (1∶200 dilution in NP-40 buffer) produced as described previously [44]. After a 3 h incubation at 25°C, the membrane was washed 3 times in NP-40 buffer and labeled with horseradish peroxidase (HRP) conjugated goat anti-rabbit IgG for 2 h at 25°C. The membrane was then washed 3 times with NP-40 buffer and developed with Pierce ECL western blotting substrate.

### Alexa Fluor 488 Labeling

Both Ply^WT^ and mutant Ply molecules were conjugated with Alexa Fluor 488 (AF488) tags in order to facilitate fluorescent detection. The conjugation reactions were carried out using the Alexa Fluor microscale protein labeling kit (Invitrogen Molecular Probes) according to the manufacturers instructions. The degree of labeling for each Ply type was optimized to be 3 AF488 tags per molecule of Ply. No significant difference was observed between the cytotoxicities of AF488-labeled Ply^WT^ and unlabeled Ply^WT^ (data not shown).

### Flow Cytometry

Adherent HCECs were detached from the culture flask with 0.1% trypsin-EDTA for 10 min, washed 2 times with PBS, and fixed in 3.7% paraformaldehyde (PFA) for 15 min at 25°C. The fixed cells were washed 3 times with PBS and suspended in PBS at a concentration of 10^6^ cells/ml. Fluorescent labeling was performed as described above. The fixed cell suspension was divided into 300 µl aliquots and labeled with either AF488-labeled Ply^WT^, mutant Ply, heat inactivated Ply, or BSA and incubated for 30 min at 37°C. After incubation, the cells were centrifuged at 500 x g for 3 min, and the supernatant was removed. The cells were then washed 3 times with PBS before being suspended in a final 300 µl of PBS and transferred to 5 ml dilution tubes. Ply surface binding was detected using a Gallios flow cytometer with Kaluza software version 1.1 (Beckman Coulter, Miami, FL). Each experimental condition was performed in triplicate with 5,000 events per experiment.

### Sucrose Density Gradient Centrifugation

Five x 10^6^ HCECs were collected and washed 3 times in Tris-buffered saline (TBS; 25 mM Tris/HCl, 140 mM NaCl, pH 7.5). TBS was removed and the cells were suspended in 1 ml of incubation buffer (25 mM Tris/HCl, 140 mM NaCl, 1 mM EDTA, 1 mM PMSF, Roche Complete Protease Inhibitor cocktail, pH 8) plus 25 µg of Ply and 5 µg of biotinylated cholera toxin β (CTxβ), which binds to the common lipid raft constituent ganglioside G_M1_ of eukaryotic cell membranes. The cell suspension was incubated on ice for 4 h after which 40 µl of 25% TX100 was added and mixed thoroughly (final concentration 1% TX100). The mixture was incubated an additional 1 h on ice. The solution was passed through a small gauge needle 20 times and then centrifuged at 10,000 x g for 5 min at 4°C. The supernatant was transferred to a new microcentrifuge tube, and 70% sucrose was added to a final concentration of 40% sucrose. The sample was layered under a 10–30% discontinuous sucrose gradient in a 5 ml ultracentrifuge tube at a ratio of 1.5∶2.5∶1. The gradient was centrifuged at 4°C for 18 h at 300,000 x g. Fractions were collected from the top in 400 µl increments. Fractions were spotted on a nitrocellulose membrane, blocked with 5% skim milk in NP-40 buffer for 1 h, and labeled with either HRP-conjugated streptavidin to detect CTxβ, or anti-Ply rabbit polyclonal serum followed by HRP-conjugated goat anti-rabbit IgG to detect Ply. The blot was visualized using Pierce ECL western blotting substrate.

### Statistical Analyses

Experimental results were analyzed using the statistical analysis system (SAS) for computers (SAS Institute, Cary, NC) version 9.2. All experimental groups were compared using a nonparametric one-way analysis of variance, and any P-value < 0.05 was considered significant.

## Results

**Figure 1:**
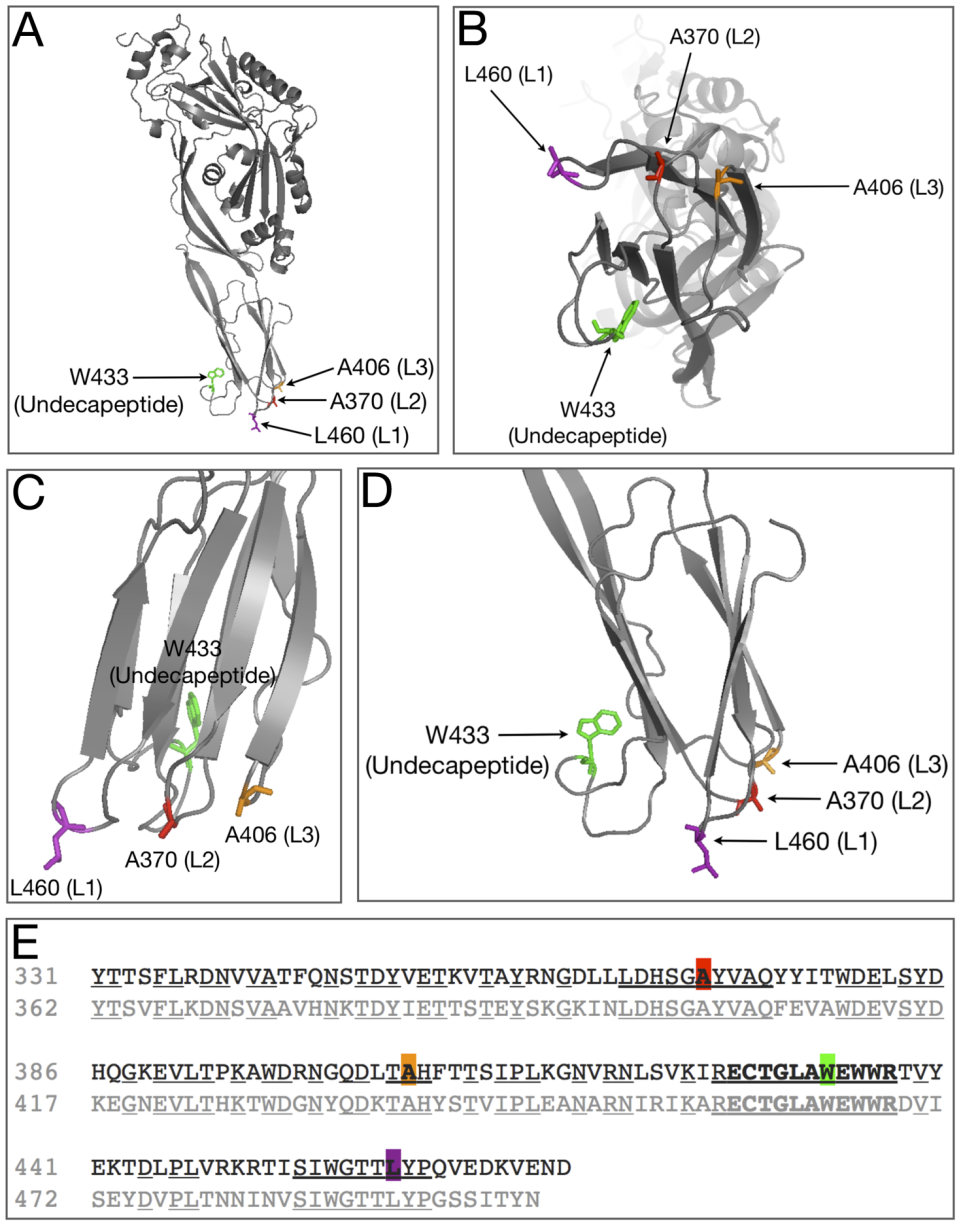
Ply Structure and Sequence Alignment. A) Ribbon diagram of Ply with the domain 4 loop residues shown at the base. B) The bottom view of domain 4 showing the relative placement of each loop. C) Zoomed in view of domain 4 and D) is a 90° rotation of domain 4. E) Sequence alignment between Ply (top line) and Pfo (bottom line), centered at the undecapeptide sequence (bold). Areas of homology between the two sequences are shown as underlined. Mutagenesis targets are shown as colored blocks.

Previous studies that focused on Pfo, a related CDC that shares 42% amino acid homology with Ply, have pinpointed several important amino acids that are involved in interacting with the lipid environment of the host membrane during initial binding [26], [45]. These residues include A401, A437, W464, and L491, and correspond to A370, A406, W433, and L460 in Ply [26], [46]. In Pfo, each of these amino acid residues are located at the tip of one of 4 loops structures found in domain 4 which extend out from the protein to interact with the host membrane. A sequence alignment of domain 4 from Ply and Pfo reveals that the loop residues from Pfo are conserved in Ply, and many of the surrounding residues around both A370 and L460 are also conserved (Figure 1E). Structural diagrams show the positions of the domain 4 loops relative to one another (Figure 1A-D). The R-groups from the highlighted amino acids of L1-L3 extend away from the interior of the molecule and presumably enter the lipophilic environment of the host cell membrane. Based on the observed homology and relative positions of each amino acid, we engineered 2 amino acid substitution mutants at the apex of each loop structure. We chose to substitute both glutamate and glycine in order to 1.) prevent the loop from entering the lipid environment of the host membrane (glutamate), and 2.) observe the effect of removing the R-group from each mutation site (glycine). We also included Ply^W433F^ since this mutation is classically studied and a wide array of information is readily available on its lytic behavior in various models. Each Ply variant was analyzed for cytotoxicity, ability to oligomerize, and ability to bind to the HCEC surface, specifically to cholesterol-rich membranes.

### The Effects of the Ply Loop Mutations on Cytolysis

**Figure 2:**
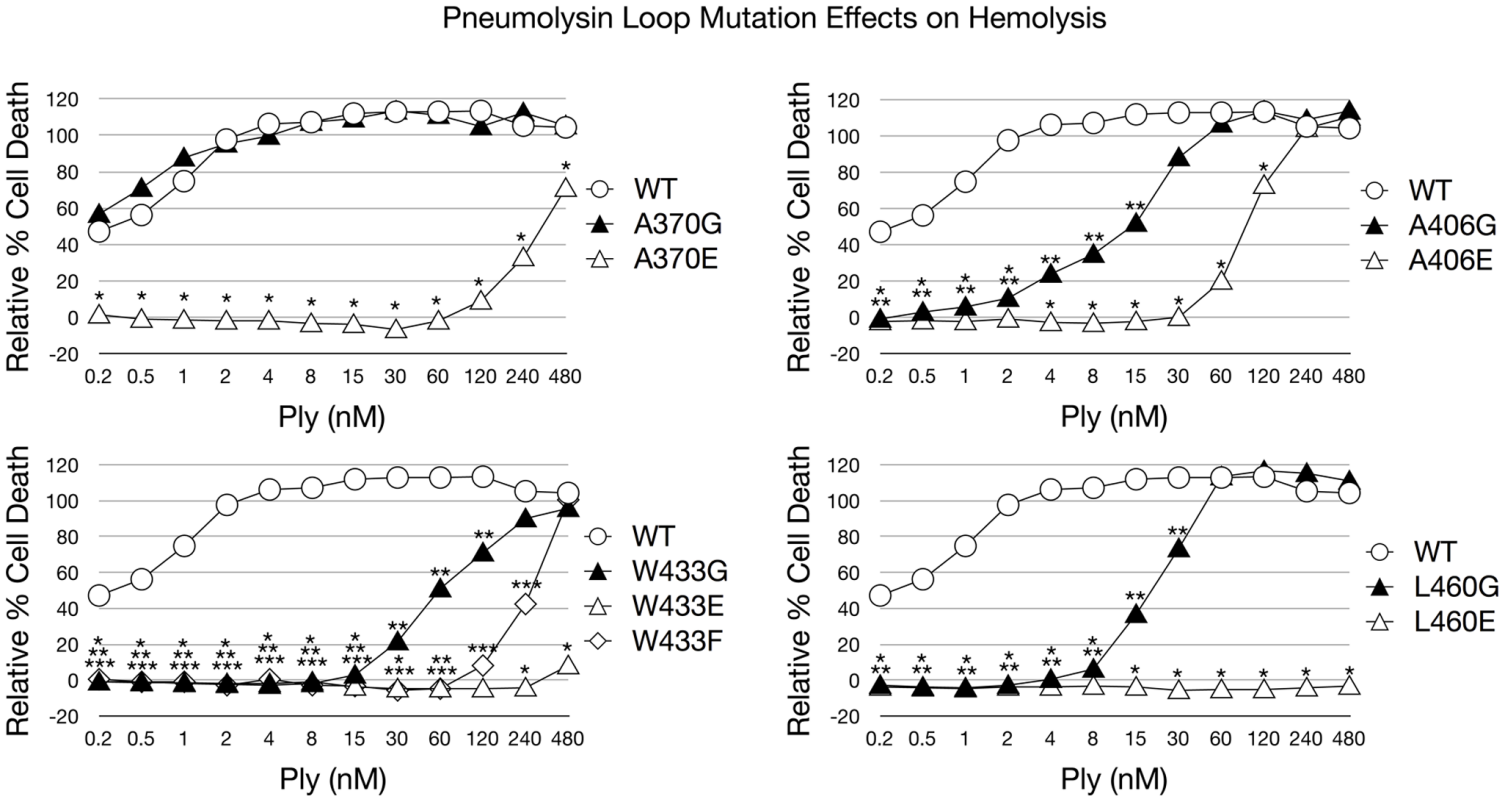
Ply Hemolytic Activity. Hemolytic activity of Ply was measured in the presence of 2% RBCs. The results are shown as relative % hemolysis. RBCs and 1% TX100 served as the positive control and represents 100% lysis. Each mutant was statistically compared to Ply^WT^ and significant differences (P<0.05) are denoted as either a single asterisk (open triangles), 2 asterisks (closed triangles), or 3 asterisks (open diamond) to account for each represented mutant. Error bars are not pictured to prevent obstruction of the data points for viewing.

**Figure 3:**
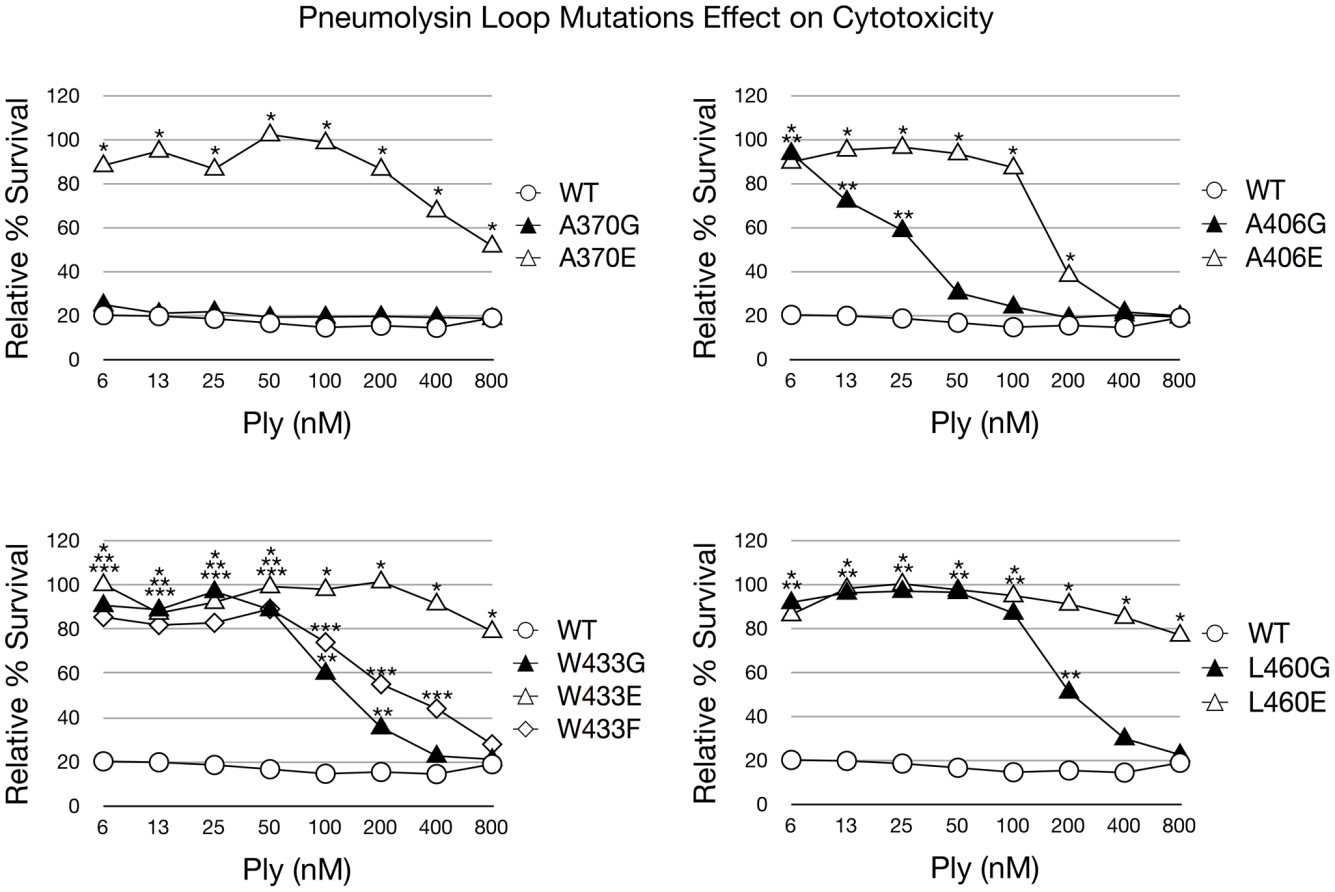
Ply Cytotoxicity on HCECs. Ply cytotoxicity on HCECs was measured by staining with calcein AM, a selective marker for healthy cells with intact membranes. Percent survival was normalized using PBS (negative) and TX100 (positive) treated cells as controls. The results are shown as % survival. Each mutant was statistically compared to Ply^WT^ and significant differences (P<0.05) are denoted as a single asterisk (open triangles), 2 asterisks (closed triangles), or 3 asterisks (open diamond) to account for each represented mutant. Error bars are not pictured to prevent obstruction of the data points for viewing.

The introduction of each amino acid substitution had a direct effect on the cytotoxicity of Ply on both RBCs and HCECs (Figures 2 and 3). It was previously reported that Ply^W433F^ has a 99% reduction in hemolytic activity, and our findings are in agreement [21]. When examining the glutamate substitution mutants, both Ply^W433E^ and Ply^L460E^ possessed the least cytolytic power, having significantly lower hemolytic activity at all concentrations examined when compared to Ply^WT^. At 480 nM, the highest Ply concentration in the hemolysis assay, Ply^L460E^ was found to be completely deficient in lytic activity. The other glutamate substitution mutants, Ply^A370E^, Ply^A406E^, and Ply^W433E^, resulted a relative cell death percentage of 71, 110, and 8 respectively. The glycine substitution mutants each exhibited a less pronounced effect than their glutamate counterparts on lytic ability, which can be expected given the polar nature of glutamate. Ply^W433G^ had the least lytic power of the glycine substitution mutants, with all hemolytic activity ceasing at 8 nM. Likewise Ply^L460G^ lost all hemolytic activity at a slightly lower concentration of 4 nM. Ply^A406G^ remained active at a concentration as low as 0.5 nM, but Ply^A370G^ showed no signs of hemolytic loss and remained as active as Ply^WT^ at all concentrations examined. The same results were found when examining the cytotoxic effect of Ply on HCECs. Based on the lytic activity observed on both RBCs and HCECs, the relative cytotoxicity of each Ply variant can be ranked from most cytotoxic to least cytotoxic in the following order: Ply^WT^, Ply^A370G^, Ply^A406G^, Ply^L460G^, Ply^W433G^, Ply^A406E^, Ply^W433F^, Ply^A370E^, Ply^W433E^, and Ply^L460E^.

### The Effects of the Ply Loop Mutations on Ply Surface Binding

**Figure 4:**
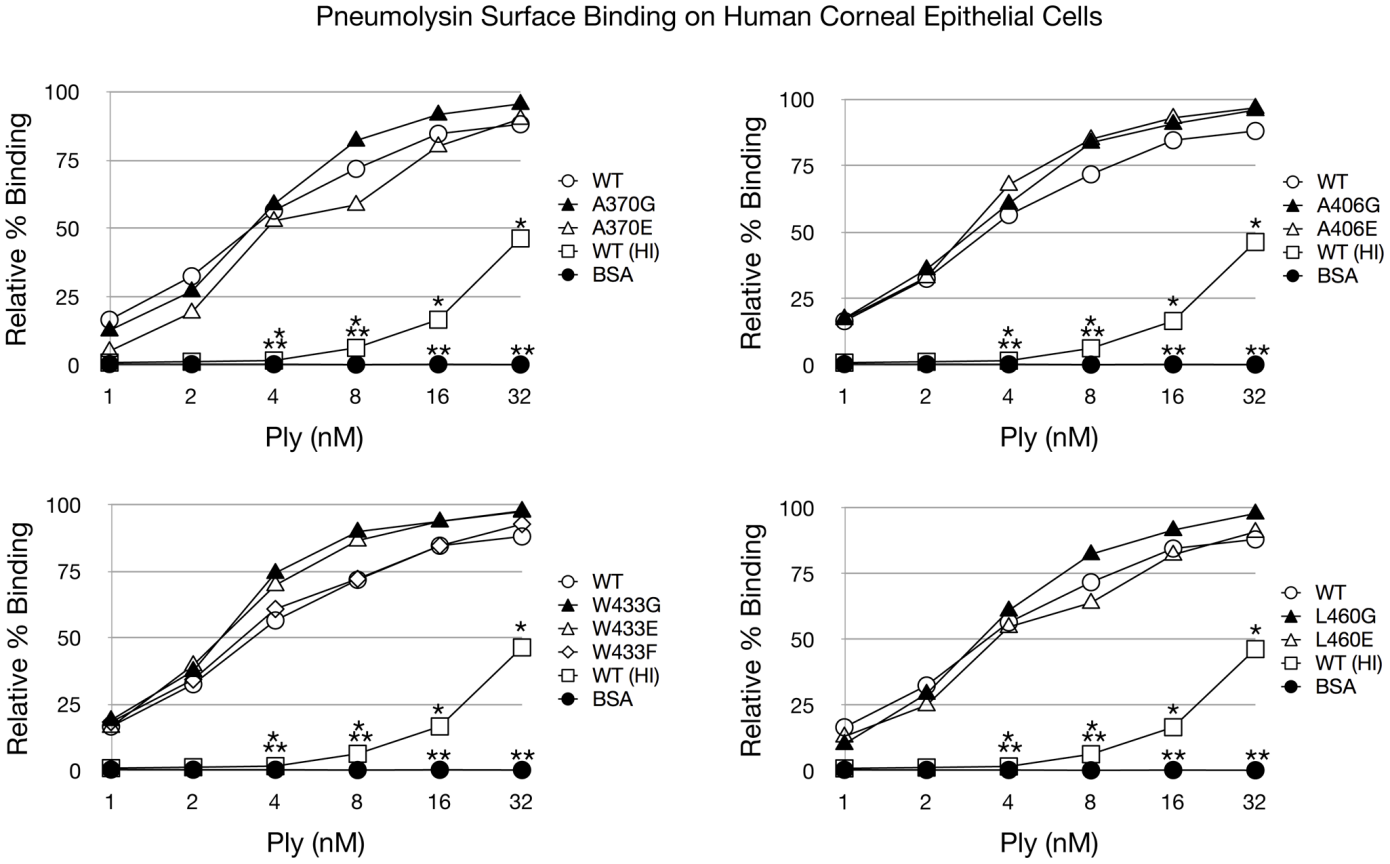
Ply Surface Binding to HCECs. Flow cytometry was used to assess surface binding to HCECs. Various concentrations of AF488 labeled Ply were incubated in the presence of 3x10^5^ fixed HCECs. AF488 labeled bovine serum albumin (BSA) and AF488 labeled heat inactivated (HI) Ply^WT^ were included as controls. Data is presented as % of cells with Ply surface binding compared to total cells. Each mutant was statistically compared to Ply^WT^ and significant differences (P<0.05) are denoted as either a single asterisk (open squares) or 2 asterisks (closed circles) to account for each group. Error bars are not pictured to prevent obstruction of the data points for viewing.

Alexa Fluor 488 labeled Ply^WT^ and all mutant variants were readily detectable as bound to the HCEC surface. Flow cytometry analysis of Ply surface binding to HCECs revealed that Ply^WT^ and all 9 Ply mutants were able to bind to the corneal cell surface, and this binding was not significantly different in any of the domain 4 loop mutants including the glutamate substitution mutants (Figure 4).

### The Effects of the Ply Loop Mutations on Ply Oligomerization

**Figure 5:**
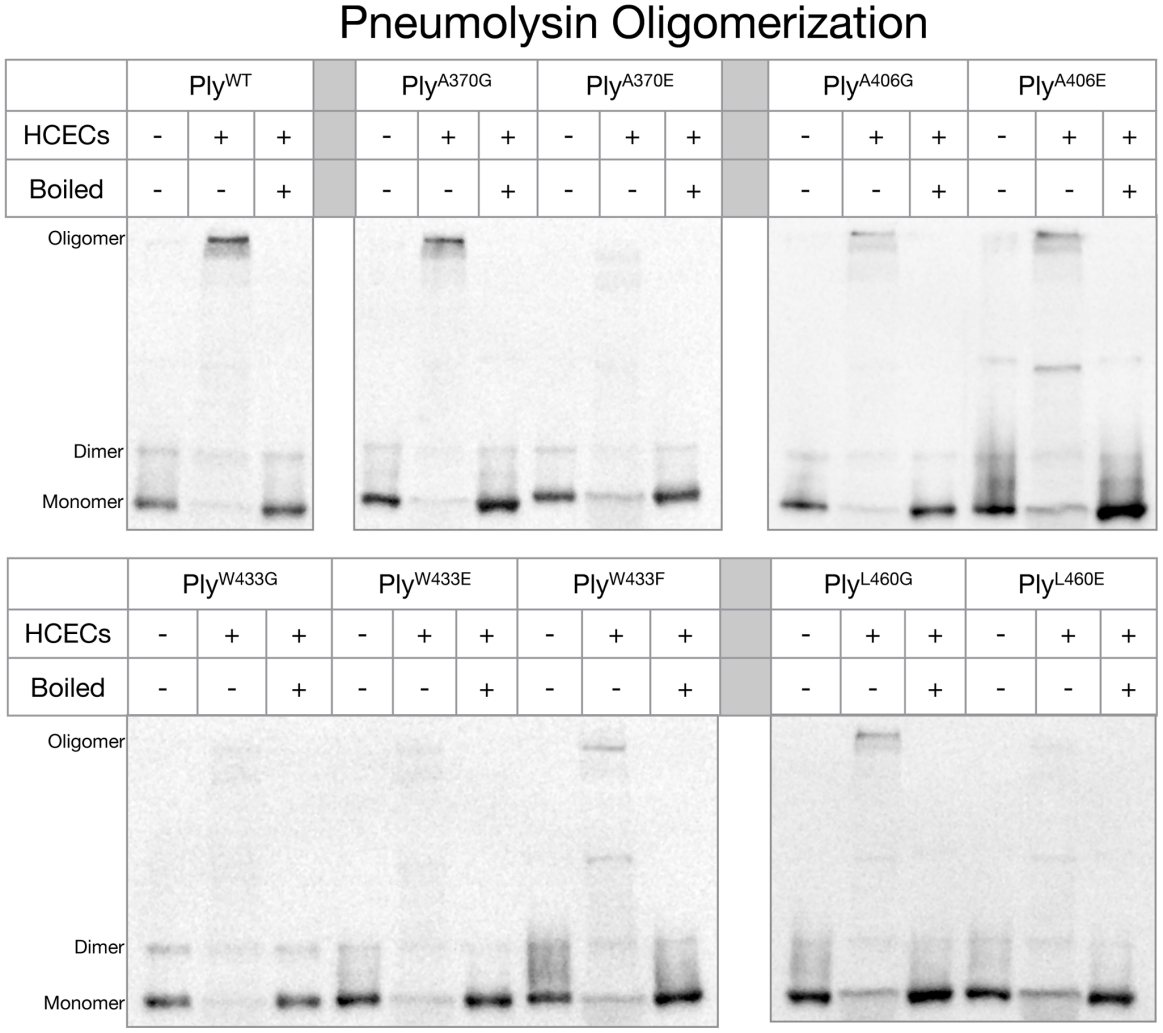
Ply Oligomerization Behavior. Ply molecules were incubated either in the presence or absence of HCECs in a total volume of 20 µl before being directly mixed with SDS loading buffer and electrophoresed through a 6% SDS polyacrylamide gel with or without boiling. The gel was blotted to a PVDF membrane, blocked in 5% skim milk, and sequentially labeled with 1° (polyclonal anti-Ply rabbit serum) and 2° (HRP conjugated goat anti-rabbit IgG) antibodies. Ply monomers are shown as 53 kDa bands along with dimers and high molecular weight oligomers.

Ply oligomeric complexes are SDS resistant and can be detected by western blot as high molecular mass bands if the samples are not boiled prior to electrophoresis [47]. After each Ply variant was incubated in the presence of HCECs, oligomeric complexes were readily detectable by western blot for Ply^WT^, Ply^A370G^, Ply^A406G^, Ply^A406E^, Ply^W433F^ and Ply^L460G^, but not Ply^A370E^, Ply^W433G^, Ply^W433E^, and Ply^L460E^ (Figure 5). Ply^WT^ and Ply^A370G^ exhibited the darkest high molecular weight oligomer band in agreement with the fact that Ply^A370G^ retained full lytic activity. Ply^A406G^, Ply^A406E^, Ply^W433F^, and Ply^L460G^ all retained their ability to oligomerize in the presence of cholesterol rich membranes although at a visibly diminished efficiency when compared to Ply^WT^. Interestingly, both Ply^A370^ and Ply^L460^ followed a similar pattern where glutamate substitution rendered both mutants unable to oligomerize, but glycine substitution allowed for the retention of oligomerization ability, albeit reduced from Ply^WT^. The A406 loop is unique in that neither substitution resulted in a loss of oligomerization. The W433 loop was only able to oligomerize with the phenylalanine substitution, which is a conservative substitution. The loss of either the W433 loop insertion into the membrane or the loss of the R-group resulted in no oligomer formation.

### The Effects of the Ply Loop Mutations on Lipid Raft Colocalization

**Figure 6:**
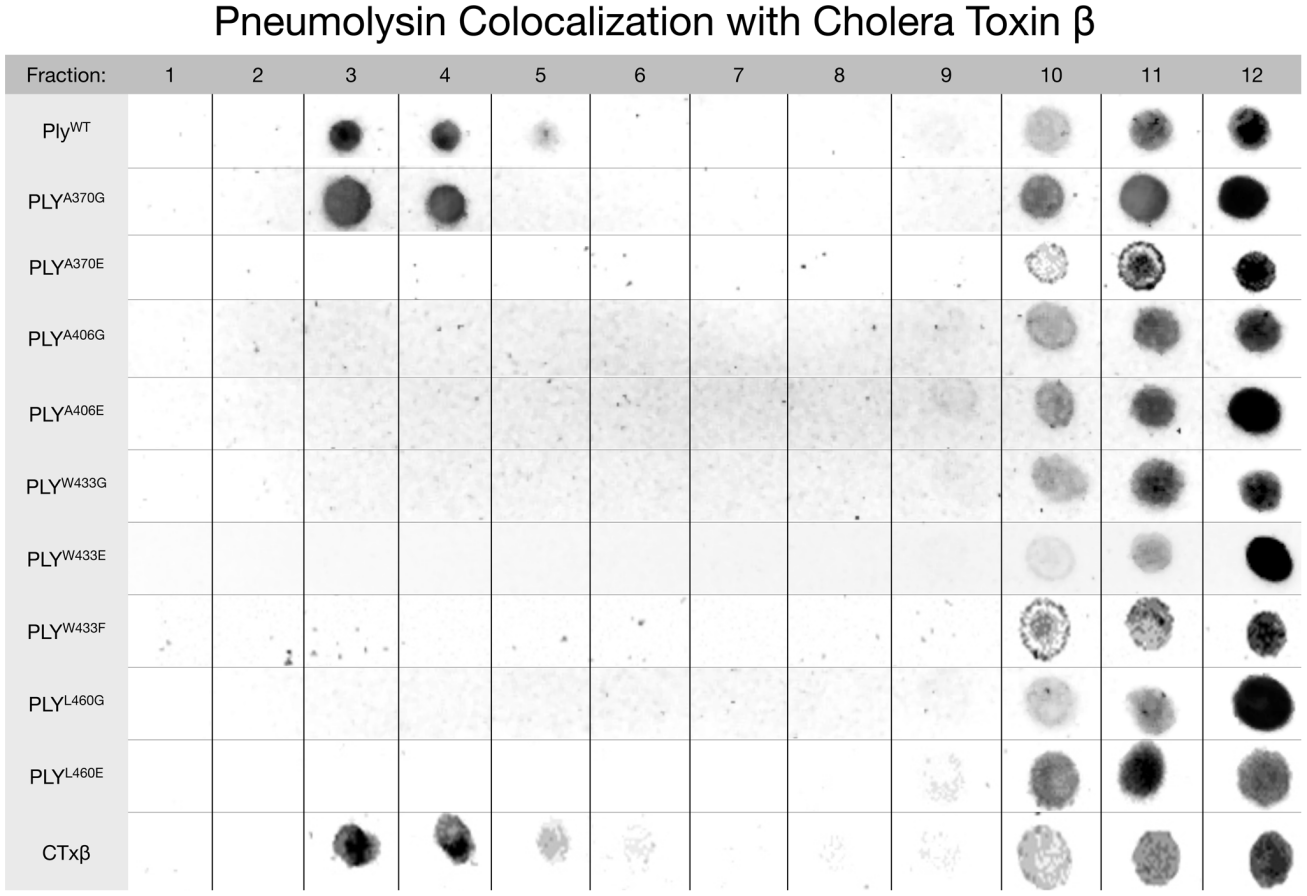
Ply Localization to Lipid Raft Microdomains. Sucrose density gradients were performed to isolate lipid raft microdomains from the phospholipid membrane and to visualize whether Ply localized with the lipid raft fractions. Gradient fractions were blotted onto a nitrocellulose membrane, and Ply or CTxβ were detected by chemiluminescence. Fractions are numbered from least dense to most dense.

We performed sucrose density gradient centrifugations with solubilized HCECs incubated with both Ply and CTxβ^Biotinylated^ in order to separate the low density lipid rafts from the high density phospholipid bilayer (Figure 6). The sucrose gradients revealed that both Ply^WT^ and CTxβ did in fact localize to the low density lipid raft fractions at the top of the sucrose gradient (fractions 3-5). However, of the 9 mutants, only Ply^A370G^ was found in the low density lipid raft fractions. The remaining mutants were only found in the high density fractions (fractions 9-12) and were unable to localize to the lipid raft fractions of the gradient.

## Discussion

The fact that 8 of the 9 mutant Ply variants exhibit a reduction in cytotoxicity when compared to Ply^WT^, but none of the mutants are deficient in HCEC surface binding indicates that the domain 4 loops are, in some capacity, involved in initiating oligomerization and/or the prepore to pore conversion. The sequence alignment of domain 4 Pfo and Ply shows nearly 75% sequence homology between the two molecules (Figure 1). The undecapeptide (W433) along with the L460 loop and the A370 loop are 100% conserved between Ply and Pfo, however, there is sequence variability when comparing the A406 loop. This lack of homology at the A406 loop may indicate that this loop is of less importance than the other more conserved loops in terms of contributing to the progression of the lytic mechanism. Additionally, substitution of glycine or glutamate at the A406 position has a smaller effect on cytotoxicity than those same mutations at any of the other loops, with the only exception being Ply^A370G^. The fact that Ply^A370G^ resulted in no reduction in cytotoxicity, but Ply^A370E^ did result in significant reduction in cytotoxicity indicates that the native alanine at position 370 is likely not involved in any direct molecular interactions with the membrane constituents, but rather simply supplies a stabilizing effect that still occurs when A370 is substituted with glycine. Additionally, the fact that Ply^A370E^ prevents oligomerization indicates that the A370 loop must interact with the membrane in order for oligomerization to occur.

Ply and Pfo as a whole have been shown to be largely conserved in structure and amino acid sequence, so findings regarding one are likely to be at least partly applicable to another. However, we have discovered some behavioral differences between Ply and Pfo with regard to surface binding. Previous research conducted by Ramachandran et al. has shown that all 4 homologous domain 4 loops of Pfo interact with the lipid environment during cholesterol binding, and Soltani et al. observed that when the loop residues of Pfo are individually mutated to aspartate, then surface binding to cholesterol containing membranes is almost completely abolished [26], [46]. Our surface binding results for Ply and HCECs are unique from the findings observed for Pfo. When the domain 4 loops of Ply are mutated to glutamate, then surface binding to HCECs is unaffected as all mutants bind with the same efficiency as Ply^WT^. This difference from the observed findings for Pfo indicates one of three possibilities: 1) aspartate and glutamate result in two different outcomes when substituted at the loop residues, 2) Pfo and Ply have different binding behaviors which react differently to the presence of a charged polar amino acid in the loops of domain 4, or 3) the observed difference is due to the use of HCECs as the target cell.

A recent study by Farrand et al. reported that the CDC cholesterol recognition motif for several CDCs including Pfo and Ply is a threonine-leucine amino acid pair found in domain 4, corresponding to T459 and L460 in Ply [34]. They found that double glycine substitutions of these residues dramatically reduced cholesterol binding on RBCs, and this threonine-leucine pair is conserved across all CDCs. Interestingly, our results indicate that when Ply^L460^ is substituted with glutamate, it still retains its ability to bind to the surface of HCECs at an undiminished capacity when compared to Ply^WT^. This binding behavior was not expected due to the previous results showing that T459 and L460 comprised the cholesterol recognition motif for Ply when exposed to cholesterol-rich liposomes. Our results indicate that it is unlikely that L460 is part of the cholesterol recognition motif of Ply when targeting HCECs, since the addition of a polar charged residue or removal of the R-group has no observed effect on surface binding to HCECs. Likewise, in addition to Ply^L460E^, flow cytometry revealed that the other glutamate substitution mutants, Ply^A370E^, Ply^A406E^, and Ply^W433E^ were also capable of binding to the surface of HCECs with no significant differences when compared to Ply^WT^. These results indicate that cholesterol recognition and binding by Ply is likely carried out not by a single loop structure, but rather a concerted effort between 2 or more of the loops.

The oligomerization behaviors of our Ply variants also yielded some unique results when compared to other CDCs. We observed that Ply^W433G^ was unable to form oligomeric complexes under our experimental conditions. However, a previous study that examined the oligomerization behavior of Ily found that Ily^W491A^, the Ily mutant corresponding to same position as Ply^W433G^, was able to form high molecular weight oligomeric complexes [33]. Interestingly, Ply^W433F^ was also found to be capable of oligomerization indicating that W433 is likely involved in a molecular interaction required for oligomerization to occur, since a conservative substitution, tryptophan to phenylalanine, resulted in the retention of oligomerization ability. The same study by Soltani et al. observed that Ily^L518D^ was able to oligomerize, although at a markedly reduced capacity when compared to Ily^WT^. Our Ply mutant with a similar mutation, Ply^L460E^, was unable to oligomerize at any detectible level. In this particular case we substituted glutamate rather than aspartate and this may account for the differing oligomerization behavior. However, Ily^A428D^, compared to the homologous Ply mutant Ply^A370E^, behaved in a similar manner where neither mutant was able to oligomerize, and likewise the same might be said for Ily^A464D^ and Ply^A406E^ [33]. The oligomerization deficiency caused by Ply^A370E^ and Ply^L460E^ indicates that these amino acids must be within the lipid membrane environment in order for oligomerization to occur on the HCEC surface. They likely function in a stabilizing role, since mutation of both of these residues to glycine did not prevent oligomerization. Therefore, the R-groups of A370 and L460 are not likely involved in specific molecular interactions that are required for the oligomerization of Ply on HCECs. The reduction in oligomerization efficiency may be due to destabilization caused by the presence of glycine rather than the native residues.

We then questioned where on the HCEC surface Ply was localizing and if there was any difference between Ply^WT^ and the loop mutants. Previous research investigating both Pfo and listeriolysin (Llo) have shown that they preferentially localized to lipid raft microdomains on the surface of human lymphoblastic cells (MOLT-4) and mouse macrophage (J774 cells) respectively [48], [49]. Specifically, Llo has been shown to cause lipid raft markers, including ganglioside G_M1_, to aggregate on the surface of J774 macrophages [48]. The same study by Gekara *et al.* discovered that lipid raft aggregation by Llo can be blocked if the toxin is pretreated with a monoclonal antibody that blocks oligomerization, but not cholesterol recognition. Therefore they proposed that oligomerization of Llo is responsible for lipid raft aggregation and may facilitate other cellular functions such as endocytosis or the initiation of intracellular signaling. Very little is known regarding Ply and whether it interacts with lipid raft microdomains on the HCEC surface. We performed sucrose density gradient centrifugations of HCEC membranes in order to separate the lipid rafts from the surrounding bilayer, after labeling the cells with CTxβ and Ply. We observed that both Ply^WT^ and CTxβ were detectable in both the low (raft) and high (non-raft) density fractions. Interestingly, all of the mutant Ply molecules were only detectable in the high density fractions except for Ply^A370G^, the only fully lytic mutant. The fact that the majority of the mutant Ply molecules were only detectable in the high density fractions indicates that although initial binding is not affected, the loop mutations do influence the ability of the molecules to localize to lipid raft microdomains on the HCEC surface. The nature of this disruption is still not fully understood. The fact that only the fully active Ply variants (Ply^WT^ and Ply^A370G^) were detected in the raft fractions indicates that either full oligomerization capability is necessary or the ability to convert the prepore to mature pore is required for the raft colocalization to occur. Of the Ply mutants that were found to be oligomerization capable (Ply^A370G^, Ply^A406G^, Ply^A406E^, Ply^W433F^, and Ply^L460G^), Ply^A370G^ is the only mutant that is as efficient as Ply^WT^ at oligomer formation and was also the only mutant that localized to the raft fractions of the sucrose gradient. Perhaps the diminished capacity to oligomerize results in an inability to colocalize with lipid rafts. The process of oligomerization may induce the formation of raft microdomains, or alternatively, lipid rafts could fill a critical role in the process of oligomerization, such as stabilization of the monomers on the cell surface. In either case, the loop mutations result in both a reduction in oligomerization and lipid raft colocalization.

In conclusion, the domain 4 loop mutations are clearly involved in the lytic mechanism of Ply since the cytotoxicity of each mutant was found to be considerably reduced as compared to Ply^WT^ (except for Ply^A370G^). None of the loop mutations prevented binding to the surface of HCECs, although Ply^A370E^, Ply^W433G^, Ply^W433E^, and Ply^L460E^ did prevent the process of oligomerization. The binding behavior of the mutants indicates that cholesterol recognition is likely not carried out by a single loop, but rather a concerted process involving multiple loops, since preventing any single loop from membrane interaction by glutamate substitution never resulted in a loss of surface binding. None of the Ply loop mutants were found to localize with the low density lipid raft fractions of HCEC sucrose density gradients (again except Ply^A370G^), where Ply^WT^ was detectable. These findings may be unique to Ply or to the interaction of Ply specifically with HCECs. Although the exact relationship between the domain 4 loops and their interaction with cholesterol and lipid rafts is not fully understood, it is apparent that lipid raft microdomains play a critical role in the lytic mechansim of Ply.
